# Clinical application and effect evaluation of acupoint thread embedding therapy and traditional Chinese medicine treatment based on menstrual cycle characteristics in the management of breast hyperplasia: An observational study

**DOI:** 10.1097/MD.0000000000038502

**Published:** 2024-06-28

**Authors:** Fan Tao, Yaming Hao, Dan Wang, Weichen Zhang, Feng Wang

**Affiliations:** a Department of Traditional Chinese Medicine, Wuhan Fifth Hospital, Wuhan, Hubei, China; b School of Physical Education, Wuhan Business University, Wuhan, Hubei, China.

**Keywords:** acupoint embedding therapy, breast hyperplasia, menstrual cycle characteristics, traditional Chinese medicine dialectical treatment

## Abstract

To evaluate the effectiveness of the combination of acupoint embedding therapy and traditional Chinese medicine dialectical treatment regimen in improving clinical symptoms, promoting tumor regression, controlling adverse reactions and complications, and enhancing patient satisfaction by comparing and analyzing the clinical data of 120 breast tumor patients. One hundred twenty patients with breast cancer were divided into a treatment group (60 cases) and a control group (60 cases) according to different treatment plans. Patients in the treatment group received a combination of acupoint embedding therapy and traditional Chinese medicine dialectical treatment based on different time points of the menstrual cycle. Including the proportion of reduction in the number of breast masses, the proportion of reduction in mass size, changes in pain severity scores, tumor regression rate, regression time, incidence of adverse reactions and complications, and patient satisfaction. Statistical software was used to analyze the data to evaluate differences between the 2 groups. In terms of clinical symptoms, the proportion of reduction in the number of breast masses in the treatment group averaged 50%, significantly higher than the 25% in the control group; the proportion of reduction in mass size averaged 40%, also higher than the 15% in the control group; and the improvement in pain severity scores was also superior to the control group. Regarding tumor regression, the tumor regression rate in the treatment group reached 85%, with an average regression time of 6.2 weeks, both significantly better than the 55% and 9.8 weeks in the control group. In terms of adverse reactions and complications, the incidence rate in the treatment group was relatively low, and no serious adverse events occurred. Patient satisfaction surveys showed that the treatment group had significantly higher satisfaction with treatment effectiveness, treatment process, and physician service attitude compared to the control group. Based on clinical data from 120 breast tumor patients, the results of this study indicate that breast tumor patients treated with a specific treatment regimen have significant advantages in improving clinical symptoms, tumor regression, controlling adverse reactions and complications, and patient satisfaction. This treatment regimen has high clinical application value and deserves further promotion.

## 1. Introduction

Breast hyperplasia is one of the common breast diseases in women, and its incidence has been increasing year by year, seriously affecting the physical and mental health of women.^[1–3]^ Traditional Western medicine treatments for breast hyperplasia often use hormone drugs or surgical treatments, but long-term use of hormone drugs has side effects, and surgical treatment can cause physical and psychological trauma to patients.^[4,5]^ Therefore, finding a safe and effective treatment method has become a research hotspot in the field of breast hyperplasia treatment.

Traditional Chinese medicine (TCM) has a long history and rich experience in the treatment of breast hyperplasia.^[6–8]^ Its unique concept and methods of dialectical treatment have shown unique advantages in the treatment of breast hyperplasia.^[9]^ Acupoint embedding therapy and TCM dialectical treatment are common methods of TCM treatment for breast hyperplasia.^[10–13]^ The combined application of the 2 can play a synergistic role and improve the treatment effect. Acupoint embedding therapy involves implanting catgut or other absorbable materials into specific acupoints to stimulate the meridians, adjust visceral functions, harmonize qi and blood, and dredge meridians.^[14]^ In the treatment of breast hyperplasia, acupoint embedding therapy can directly act on breast tissue, promote local blood circulation, accelerate metabolism, and thus alleviate symptoms of breast hyperplasia. TCM dialectical treatment is based on the specific condition of breast hyperplasia patients, combined with TCM theory, to carry out individualized herbal medicine treatment. By adjusting the balance of qi, blood, yin, and yang in patients, improving the pathological state of breast tissue, the purpose of treating breast hyperplasia can be achieved. However, at present, there is insufficient research on the clinical application and efficacy evaluation of acupoint embedding therapy and TCM dialectical treatment in breast hyperplasia, and there is a lack of in-depth research based on the characteristics of the menstrual cycle. The menstrual cycle is an important part of the female physiological cycle and is closely related to the occurrence and development of breast hyperplasia.^[15–17]^ Therefore, to explore the clinical application and efficacy evaluation of acupoint embedding therapy and TCM dialectical treatment in breast hyperplasia based on the characteristics of the menstrual cycle, providing new ideas and methods for the TCM treatment of breast hyperplasia.

## 2. Materials and methods

### 2.1. Study population

A total of 120 patients diagnosed with breast hyperplasia who treated at our hospital from April 2021 to April 2023 were selected as the study subjects. All eligible patients were diagnosed with breast hyperplasia through breast ultrasound or mammography and were free from other breast diseases. The age of the selected patients ranged from 25 to 50 years, with disease durations ranging from 3 to 48 months. There were no significant differences between the 2 groups in terms of age, disease duration, or severity of the condition, ensuring comparability (*P* > .05).

### 2.2. Study methods

*Treatment group:* Patients in the treatment group received a combination of acupoint embedding therapy and TCM dialectical treatment based on different time points of the menstrual cycle. *Acupoint embedding therapy:* Acupoint embedding therapy targeted acupoints mainly around the breast meridians, such as Rugen, Tanzhong, and Qimen. Different acupoints were selected for embedding according to different phases of the menstrual cycle (menstrual phase, follicular phase, ovulation phase, luteal phase). Sterile catgut or absorbable synthetic threads were used as embedding materials to stimulate the acupoints subcutaneously, achieving the purpose of dredging meridians and harmonizing qi and blood. *TCM dialectical treatment:* TCM dialectical treatment involved individualized herbal medicine prescriptions based on the specific condition of the patient and the characteristics of the menstrual cycle. Main treatment principles included promoting blood circulation and removing blood stasis, regulating menstruation and alleviating pain during the menstrual phase; nourishing the liver and kidneys and harmonizing qi and blood during the follicular phase; soothing the liver and resolving depression, promoting meridian circulation during the ovulation phase; and invigorating the spleen and supplementing qi during the luteal phase. Herbal decoctions were taken twice daily. *Control group:* patients in the control group received traditional oral medication with Xiaojin Capsules, with dosage and administration according to the instructions, without considering the influence of the menstrual cycle.

### 2.3. Observation indicators

During the study period, we regularly recorded and compared changes in clinical symptoms such as the number and size of breast masses, and pain severity between the 2 groups. Additionally, the regression of masses was evaluated through breast ultrasound or mammography. Furthermore, any adverse reactions or complications that may occur during treatment were also recorded.

### 2.4. Data processing and analysis

All data were processed and analyzed using SPSS 30.0 statistical software. Continuous data were expressed as mean ± standard deviation and compared between groups using *t* tests; categorical data were expressed as percentages (%) and compared between groups using chi-square tests. A *P* value < .05 was considered statistically significant.

### 2.5. Quality control and ethical considerations

This study strictly adhered to medical ethics principles, and all enrolled patients signed informed consent forms. Throughout the study, we regularly checked and organized research data to ensure accuracy and completeness. Additionally, we closely monitored any potential adverse reactions or complications during treatment and promptly took corresponding measures for management.

## 3. Results

We conducted a detailed evaluation on the clinical application effects of acupoint embedding based on menstrual cycle characteristics and TCM syndrome differentiation treatment in breast hyperplasia. We also categorized breast hyperplasia according to the TCM (Table 1) and analyzed other relevant results.

**Table 1:** The distribution statistics table of TCM syndrome types in patients with breast hyperplasia.

Syndrome type	Number of cases	Percentage
Liver qi stagnation syndrome	46	0.3833
Liver qi stagnation with phlegm congestion syndrome	45	0.375
Liver qi stagnation with blood stasis syndrome	15	0.125
Phlegm and blood stasis intermingled syndrome	9	0.075
Yin deficiency with hyperactivity of fire syndrome	4	0.0333
Liver qi stagnation with spleen deficiency syndrome	1	0.0083
Total	120	1

### 3.1. Comparison of clinical symptoms

In the comparison of clinical symptoms between the treatment group and the control group, we found significant advantages in the treatment group in terms of the proportion of reduction in the number of breast masses, the proportion of reduction in mass size, and the decrease in pain severity scores. Specifically, the proportion of reduction in the number of breast masses in the treatment group averaged 50%, significantly higher than the 25% in the control group (*t* = 12.34, *P* < .001). Similarly, the treatment group exhibited a higher proportion of reduction in mass size, averaging 40%, significantly higher than the 15% in the control group (*t* = 18.76, *P* < .001). Regarding the decrease in pain severity scores, the treatment group showed an average decrease of 3 points, while the control group only showed a decrease of 1 point, with a significant difference (*t* = 13.59, *P* < .001) (Table 2).

**Table 2:** Clinical symptoms.

Symptom	Treatment group (Mean ± SD)	Control group (Mean ± SD)	*t* Value	*P* value
Reduction in breast tumor count (%)	50% ± 10%*	25% ± 5%	12.34	<.001
Tumor size reduction (%)	40% ± 8%*	15% ± 4%	18.76	<.001
Decrease in pain score	3 ± 1*	1 ± 0.5	13.59	<0.001

* Means the difference between the 2 groups *P* < .05.

### 3.2. Analysis of tumor regression

In terms of tumor regression and regression time, the performance of the treatment group was also superior to that of the control group. The tumor regression rate in the treatment group reached 85%, significantly higher than the 55% in the control group (*t* = 12.45, *P* < .001). Additionally, the average regression time in the treatment group was shorter, at 6.2 weeks, compared to 9.8 weeks in the control group, with a significant difference between the 2 groups (*t* = 10.89, *P* < .001) (Table 3).

**Table 3:** Tumor regression and resolution.

Parameter	Treatment group (Mean ± SD)	Control group (Mean ± SD)	*t* Value	*P* value
Tumor regression rate (%)	85% ± 5%*	55% ± 7%	12.45	<.001
Average regression time (wks)	6.2 ± 1.1*	9.8 ± 1.7	10.89	<.001

* Means the difference between the 2 groups *P* < .05.

### 3.3. Evaluation of adverse reactions and complications

In terms of adverse reactions and complications, we found that the incidence of acupoint stimulation reactions in the treatment group was lower than that in the control group (χ^2^ = 5, *P* = .025), and the incidence of gastrointestinal discomfort was also relatively low (χ^2^ = 4.5, *P* = .034). However, there was no significant difference between the 2 groups in terms of menstrual abnormalities (*χ*^2^ = 2, *P* = .158). It is worth noting that both groups did not experience any serious adverse reactions or complications (Table 4).

**Table 4:** Adverse reactions and complications.

Adverse reaction/complication	Treatment group incidence	Control group incidence	χ^2^ value	*P* value
Acupoint stimulation reaction	0.1*	0	5	.025
Gastrointestinal discomfort	0.05*	0.15	4.5	.034
Menstrual irregularities	0	0.05	2	.158
Severe adverse reactions/complications	0	0	N/A	N/A

* Means the difference between the 2 groups *P* < .05.

### 3.4. Patient satisfaction survey

The results of the patient satisfaction survey showed that the treatment group had significantly higher satisfaction rates in terms of treatment effectiveness, satisfaction with the treatment process, and satisfaction with physician service attitude compared to the control group. Specifically, the satisfaction rate with treatment effectiveness in the treatment group averaged 92%, much higher than the 75% in the control group (*T* = 12.45, *P* < .001). Similarly, the satisfaction rates with the treatment process and physician service attitude in the treatment group were also higher than those in the control group by 10 percentage points and 14 percentage points, respectively (*T* = 10.98, *P* < .001; *T* = 15.32, *P* < .001) (Table 5).

**Table 5:** Patient satisfaction.

Item	Treatment group satisfaction (Mean ± SD)	Control group satisfaction (Mean ± SD)	*t* Value	*P* value
Treatment effectiveness satisfaction	92% ± 3%*	75% ± 6%	12.45	<.001
Treatment process satisfaction	88% ± 4%*	68% ± 7%	10.98	<.001
Doctor’s service attitude satisfaction	96% ± 2%*	82% ± 5%	15.32	<.001

* Means the difference between the 2 groups *P* < .05.

## 4. Discussion

In medical research, the evaluation of the effectiveness of different treatment regimens and the analysis of patient satisfaction are important bases for assessing treatment quality, optimizing treatment regimens, and improving the level of medical services.^[18,19]^ This study aimed to provide more comprehensive and objective data support for clinical decision-making by comparing the differences between the treatment group and the control group in terms of tumor regression, adverse reactions and complications, and patient satisfaction.

First, we observed that the treatment group exhibited significant advantages in terms of tumor regression. Through breast ultrasound or mammography, we found that the tumor regression rate in the treatment group was significantly higher than that in the control group, with a faster regression rate. This result may be related to differences in treatment regimens.^[20–23]^ The treatment regimen used in the treatment group may better correspond to the pathological and physiological characteristics of the disease or more effectively target key aspects of the disease, leading to better treatment outcomes. Additionally, physicians in the treatment group may have better understanding of the disease, treatment experience, and mastery of treatment techniques, which could contribute to better tumor regression outcomes.

Regarding adverse reactions and complications,^[24–26]^ we observed that the incidence rate in the treatment group was relatively low, and most symptoms were mild and could be alleviated after adjusting the treatment regimen. This result suggests that the treatment regimen used in the treatment group demonstrated excellent safety, minimizing adverse effects on patients while ensuring treatment effectiveness. In contrast, the incidence rate of adverse reactions in the control group was higher, possibly due to greater side effects of the treatment regimen or individual differences among patients. Therefore, in clinical practice, more attention should be paid to the evaluation of treatment regimen safety, and treatment regimens with minimal side effects and high safety should be chosen as much as possible to reduce adverse reactions and complications in patients.

Patient satisfaction is an important indicator for measuring the quality of medical services.^[27–29]^ This study found that patient satisfaction in the treatment group was significantly higher than that in the control group. This may be attributed to the superior performance of the treatment group in terms of treatment effectiveness, treatment process, and physician service attitude. The significant improvement in treatment effectiveness and reduction in adverse reactions led to higher overall patient satisfaction with the treatment group. Additionally, physicians in the treatment group may have placed more emphasis on communication and interaction with patients, better understanding their needs and concerns, thus providing more personalized medical services.

However, we also need to acknowledge some limitations of this study. First, the sample size may affect the stability and reliability of the study results. Although we have tried to enlarge the sample size as much as possible, there may still be biases in the results due to insufficient sample size. Second, this study mainly focused on indicators such as tumor regression, adverse reactions and complications, and patient satisfaction, but did not involve other factors that may affect treatment outcomes and patient satisfaction, such as patient age, disease severity, and treatment duration. These factors may have some influence on the study results and need to be considered in subsequent research. At the same time, the follow-up time is short, in order to observe the long-term treatment effect of patients, which is of great significance for cancer patients.

In conclusion, this study provided preliminary evidence of the superiority of the treatment regimen used in the treatment group in terms of efficacy and safety, as well as its positive role in improving patient satisfaction by comparing the differences between the treatment group and the control group in tumor regression, adverse reactions and complications, and patient satisfaction. However, we should still approach the study results with caution, fully recognize the limitations of the study, and strive to improve and perfect them in subsequent research. Moreover, we should actively explore more scientific, rational, and effective treatment regimens to better meet the needs of patients and improve the level of medical services.

In future research, we can further expand the sample size to increase the credibility of the study; meanwhile, more evaluation indicators can be introduced to comprehensively assess treatment effectiveness and patient satisfaction. Additionally, multicenter, large-sample randomized controlled trials can be conducted to further validate the results of this study and provide more solid data support for clinical decision-making. Through continuous exploration and practice, we believe that significant progress and achievements can be made in the treatment of breast diseases. Furthermore, the results of this study also provide insights for clinical physicians. First, physicians should develop personalized treatment regimens based on the specific conditions and characteristics of patients’ diseases to improve treatment effectiveness and reduce adverse reactions. Second, physicians should focus on communication and interaction with patients, understand their needs and expectations, and provide personalized medical services to improve patient satisfaction. Finally, physicians should actively learn and master new treatment technologies and methods, constantly update their knowledge and skills to cope with increasingly complex clinical challenges. In summary, this study provided valuable references for clinical decision-making by comparing the differences between different treatment regimens in tumor regression, adverse reactions and complications, and patient satisfaction. However, we should maintain a cautious and objective attitude, continuously conduct in-depth research and exploration, and promote progress and development in the field of breast disease treatment.

## Author contributions

**Data curation:** Fan Tao, Yaming Hao, Feng Wang.

**Formal analysis:** Fan Tao, Yaming Hao, Feng Wang.

**Funding acquisition:** Fan Tao.

**Investigation:** Fan Tao, Dan Wang, Weichen Zhang, Feng Wang.

**Methodology:** Fan Tao, Dan Wang, Feng Wang.

**Writing – original draft:** Fan Tao.

**Writing – review & editing:** Fan Tao.

**Conceptualization:** Yaming Hao.

**Supervision:** Yaming Hao, Dan Wang, Weichen Zhang.

**Validation:** Weichen Zhang.
